# Fibroblast dynamics during mammary oncogenesis: senescence, Wnt9a and beyond

**DOI:** 10.1038/s44318-025-00446-9

**Published:** 2025-05-01

**Authors:** Viktoria Boeker, Raghu Kalluri

**Affiliations:** 1 Department of Cancer Biology, University of Texas MD Anderson Cancer Center, Houston, TX USA; 2 Department of Bioengineering, Rice University, Houston, TX USA; 3 Department of Molecular and Cellular Biology, Baylor College of Medicine, Houston, TX USA; 4 Department of Pathology, University of Texas Medical Branch, Galveston, TX USA

**Keywords:** Cancer, Cell Adhesion, Polarity & Cytoskeleton, Development

## Abstract

Recent temporal profiling of stromal fibroblast populations in the healthy and cancerous mouse mammary gland uncovers Wnt9a as a senescence inducer in cancer-associated myofibroblasts.

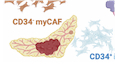

## Teaser

Mammary gland fibroblasts are highly plastic and transition through distinct states during development and disease. A new study by Pascual et al (2025) provides a comprehensive single-cell atlas of murine mammary stromal cell hierarchies, revealing dysregulation of progenitor-driven fibroblast lineages in breast cancer and a senescence role of Wnt9a in cancer-associated myofibroblasts.

The outstanding history of fibroblast research started with their description by Rudolf Virchow in 1858 in his cellular pathology books in Berlin, Germany. Since then, prodigious research efforts have been made to unravel the diverse, context-specific functions of these multifaceted stromal cells (Kalluri, 2016; Chen et al, 2021). Fibroblasts are central to shaping tissue architecture and function through extracellular matrix (ECM) remodeling, paracrine signaling, and mechano-signal transduction. In the mammary gland, these mesenchymal cells not only support epithelial morphogenesis but also contribute to cancer initiation and progression. However, their dynamic transitions, hierarchical structure, and fate decisions across physiological and pathological contexts have remained poorly characterized. Which cancer-associated fibroblasts (CAFs) function as tumor-restraining or -promoting remains an active area of scientific investigation.

**Figure 1:**
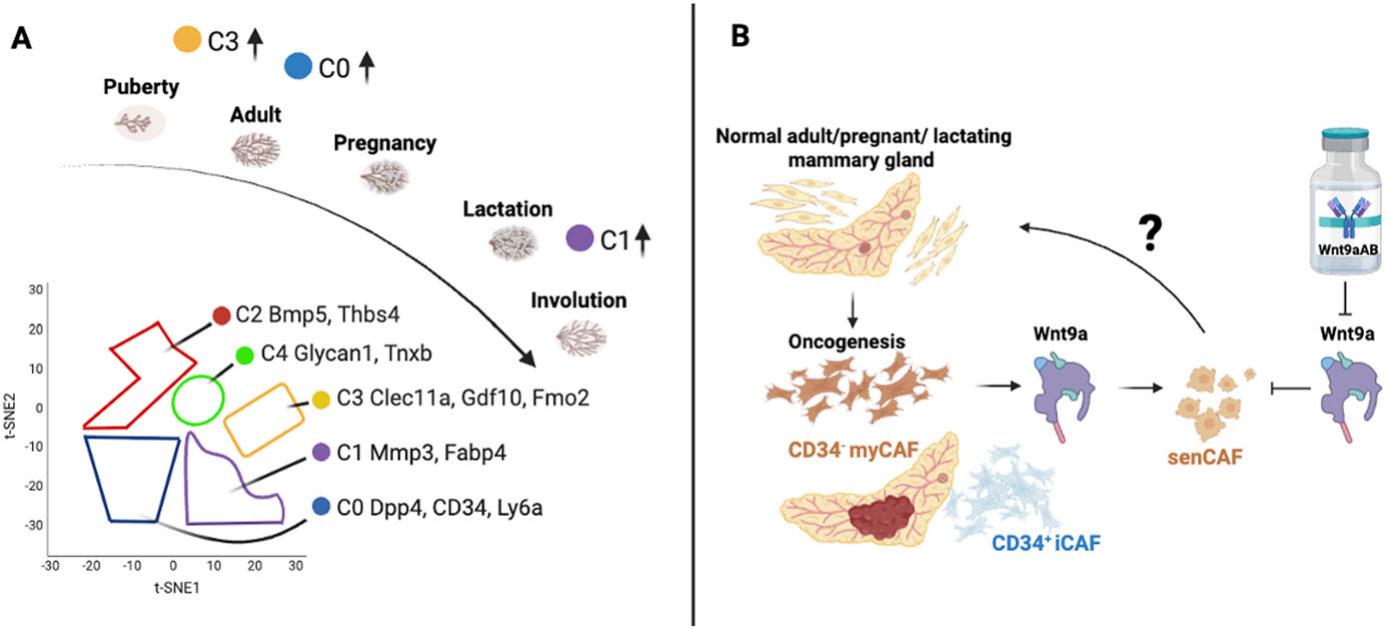
Single-cell atlas of murine mammary stromal fibroblast hierarchies. (**A**) Gene signatures of fibroblast clusters after lineage analysis during postnatal mammary development; the clusters C0-C4 revealed specific gene signatures whereby C3 was enriched in puberty, C0 during adulthood and pregnancy, and C1 during lactation and involution. Adapted from Pascual et al, (2025) (**B**). (**B**) Identification of Wnt9a as a regulator of cellular senescence in CD34^-^ myofibroblast CAFs (myCAFs) during mammary oncogenesis. CRISPR/Cas9-KO models suggest that senescence in CD34^-^ myCAFs is potentially reversible. Figure created using Biorender (https://biorender.com/).

Previous studies have shown that progressive changes in the composition of the tumor stroma drive invasive breast cancer (Risom et al, 2022). In this issue, Pascual et al (2025) apply single-cell RNA sequencing and fate-mapping to generate a high-resolution temporal and spatial atlas of mammary fibroblasts spanning postnatal development through tumorigenesis. They profiled over 45,000 stromal cells across six key developmental stages: puberty, adulthood, pregnancy, lactation, early involution, and late involution, which divided into five dominant clusters (C) (Fig. 1A). The C0 cluster contains fibroblasts expressing dipeptyl peptidase 4 (*Dpp4*), which encodes *Cd26*, stem cell markers such as *Cd34* and lymphocyte antigen 6 (*Ly6a*). The C1 cluster localized matrix metalloproteinase 3 (*Mmp3*) and the fatty acid binding protein 4 (*Fabp4*), and fibroblasts with expression of these genes were most prominent in the involution stage. A novel subset of fibroblasts was identified as present in the C2 cluster, defined by bone morphogenetic protein 5 (*Bmp5*) and thrombospondin 4 (*Thbs*). The C3 cluster was enriched in puberty with c-type lectin family (*Clec11a*), growth differentiation factor 10 (*Gdf10*), and flavin-containing monooxygenase 2 (*Fmo2*) associated fibroblasts. These genes might be relevant for the growth of adipocytes and ductal morphogenesis. The C4 cluster shares similarities with clusters C0 and C1, but additionally contained cell adhesion-related gene *Glycan 1* and the glycoprotein *Tnxb containing fibroblasts*. In general, *Pdgfra*+ fibroblasts were present consistently across various stages of postnatal morphogenesis.

CD34^hi^ mesenchymal progenitors give rise to multiple transcriptionally distinct fibroblast states, including adipogenesis-regulatory cells (Aregs), ECM-producing lobular fibroblasts, and rare cycling progenitors. These populations were differentially enriched and regulated in response to hormonal cues, particularly during pregnancy, which saw a surge in CRABP1^+^ fibroblasts characterized by active-matrix remodeling. In invasive lobular breast cancer and triple-negative breast cancer, CAFs likely originate from CD26^+^ and CD26^-^ normal fibroblast populations, giving rise to inflammatory CAFs (iCAFs) and myofibroblast CAFs (myCAFs) (Houthuijzen et al, 2023).

Other single-cell profiling studies involving primary and paired metastatic lymph nodes from breast cancer patients revealed an enrichment of the PLA2G2A + CAF subtype, which promotes immune infiltration (Liu et al, 2022). Dll1-mediated Notch signaling driving the crosstalk between CAFs and cancer cells promotes radioresistance in breast cancer (Nandi et al, 2022). The immune system’s influence by CAFs is underscored by another study investigating radioresistance, which found paracrine IL-6 signaling as a causal factor for recruitment of fibroblasts (Guo et al, 2023). In the study by Pascual et al (2025), the fibroblast landscape was dramatically altered in mammary tumors. During tumor progression, the CD34^hi^ progenitor pool was exhausted, and fibroblast identity shifted towards a CD34^lo^ myofibroblastic cancer-associated phenotype. These CD34^lo^ CAFs exhibited Hedgehog (Hh) pathway-related genes and ECM-related pathways. While CD34^hi^ CAFs were identified to be inflammatory. Interestingly, a senescent transcriptional signature, marked by increased expression of *Cdkn2a* (which encodes p16), was exclusively expressed in CD34^-^ myCAFs. Other senescent-associated genes in these CAFs were *Cdkn1a* and *Cxcl14*. In the Wnt1-driven and Brca2/Trp53-deficient mouse model, CD34^−^ CAFs also expressed high levels of *Lrrc15*. This senescence signature was previously described in pancreatic ductal adenocarcinoma (PDAC) associated CAF subsets, which was purported to accelerate PDAC and its immunosuppressive microenvironment (Belle et al, 2024).

Likewise, in a mouse model for breast cancer, senescent CAFs (senCAFs) were observed to secrete ECM, limiting natural killer cell (NK) cytotoxicity, and promoting tumor growth (Ye et al, 2024). After genetic or pharmacologic elimination of senCAFs, NK cells regained their killing ability, and tumor growth was impaired. Lineage tracing further revealed that senCAFs and myCAFs arise in parallel, sharing a common progenitor but diverging in their final fates.

A key mechanistic insight emerged in the Pascual study, when Wnt9a was identified as a driver of CAF senescence. The group compared normal breast tissues, hyperplastic breast tissues, and breast tumors. A PANTHER analysis of identified gene sets uncovered that Wnt9a expression was enriched in tumor-associated senCAFs. Using CRISPR/Cas9 editing in primary CD34^-^ myCAFs to delete Wnt9a, a suppression of senescence-associated genes and β-gal+ activity was achieved. Simultaneously, the senescence markers p21 and p16 were decreased in Wnt9a-supressed fibroblasts. These results highlight Wnt9a as inducing stromal vulnerability, with implications for reversing or modulating tumor-promoting senescence phenotype.

Overall, Pascual’s study (2025) provides new insights into fate dynamics of fibroblasts and identifies senescence as a determinant of CAFs-mediated promotion of breast cancer. But it also leaves us pondering about other questions, such as: Are senescent CAFs reversible? What cues govern progenitor exhaustion in the tumor microenvironment? Could Wnt9a inhibition synergize with current immunotherapies or stroma-targeting agents? In this regard, previous studies have shown that targeted immunotherapy against distinct CAFs can overcome treatment resistance in refractory HER2^+^ breast tumors, and that targeting MCL-1 in breast CAFs reverses their myofibroblast phenotype and pro-invasive properties (Bonneaud et al, 2022; Rivas et al, 2022). Future work integrating spatial transcriptomics, epigenetic profiling, and functional perturbation will be essential to further map the temporal transitions and plasticity of mammary stromal cells. Importantly, the report of Pascual et al, (2025) underscore the potential therapeutic promise of targeting CAFs to disrupt cancer-supportive niches and restore homeostatic tissue regulation.
